# Dissertation on the Treatment of Syphilis by the Iodide of Potassium

**Published:** 1845-10

**Authors:** 


### ART. XVIII.

1. *De Syphilide Kali hydriodico tractata, Dissertatio quam pro licentia summos in medicina honores rite capessendi publico eruditorum examini subjicit auctor* MARTINUS HASSING.—*Hauniæ*, 1845.

*Dissertation on the Treatment of Syphilis by the Iodide of Potassium.* By MARTIN HASSING.—*Copenhagen*, 1845. 8vo, pp. 92.

2. *Observations pratiquès sur le traitement des Maladies Syphilitiques par l'Iodine de Potassium.* Par le Docteur L. P. A. GAUTHIER.—*Paris et Lyon*, 1845.

*Practical Observations on the Treatment of Syphilitic Diseases by the Iodide of Potassium.* By Dr. GAUTHIER.—*Paris and Lyons*, 1845. 8vo, pp. 100.

AFTER some general observations on the modern improved methods of treating syphilis, the authors of both the works before us commence by giving a historical sketch of the introduction of the iodide into practice. The Frenchman, as usual, claims the honour for his own countrymen, Richond Desbrus, and M. Eusèbe de Salle, who used it in 1823, and M. Lallemand in 1826. These gentlemen, however, employed iodine internally, and frictions of the iodide for buboes and chronic enlargements of the testicles. The German states that Bichler, in 1822, used the iodide locally in a case of indurated bubo, thus preceding Richond. Both authors, however, agree in ascribing the credit of introducing the iodide as an *internal* remedy in *secondary* syphilis to Dr. Wallace of Dublin, and Dr. Williams of St. Thomas's hospital. Dr. Williams read his well-known paper at the College of Physicians in 1834. Ricord did not take up the inquiry until 1839, when he recommended the iodide in the tertiary cases, and in 1840, in cases of ulceration of the fauces. Dr. Gauthier, who is physician of one of the hospitals in Lyons, commenced its use in 1841. Ebers of Breslau, in 1836, was one of its earliest supporters in Germany, and Dr. Kluge has since used it extensively in the Hospital of Charity at Berlin. We have given these dates, as it is of some interest to trace the history of a medicine which, almost unknown ten years since, is now generally used throughout England, France, and Germany, and is daily becoming more employed as it is better known and appreciated. Dr. Hassing gives a long statement of the authors of each country in succession, and of the various circumstances in which they found the remedy useful, and proceeds to give an account of the views of Wallace and Ricord, quoting both freely. He then examines the value of the medicine statistically, basing his observations upon 250 cases observed in the medical section at Copenhagen between July 1838, and August 1839. All the patients were treated alike on low diet, aud the remedy was given to all in the same form. Two drachms of the iodide were dissolved in eight ounces of water, and half an ounce of this solution (7½ grains,) given three times a day. All the patients were continued for some time after cure under observation—almost all belonged to the lower class of society—and in the rare cases in which relapse occurred, the same mode of treatment was repeated.

Of the 250 patients, 68 were men, 181 women, and 1 infant, the great majority between 20 and 40 years of age. To show the kind of life the patients followed, Dr. Hassing states that 69 were maid-servants, 55 prostitutes, 32 married and 17 single women, 9 widows; 32 mechanics, 21 workmen, 6 seamen, and 9 of other professions. Of these 145 were perfectly cured, 49 imperfectly, some traces of disease remaining, and in 56 no effect was produced. The mean duration of treatment was 38 days. The shortest period at which relapse occurred was about six weeks, the longest 4 years and 9 months, the mean about 11 months.

The iodide in all the cases of primary affections in which it was employed was without effect. It exercised very little power in cases of bubo. In 20 cases of flat condylomata, 8 of which were on the anus, 10 on the genitals, and two in both situations, 7 were cured, 4 benefited, and in 9 the iodide produced no effect. The author, however, ascribes much benefit to rest and cleanliness, and concludes that the medicine does not exercise much power over this affection, whether primary or consecutive. In 49 cases of maculæ, squamæ, and papulæ of the skin, 12 being men and 37 women, 26 were cured, 9 benefited, and in 14 no effect appeared. The mean duration of their treatment was 48 days, and the author concludes that in this series of affections the iodide is a less efficacious remedy than mercury, but that the two may be skilfully combined with advantage. Of 47 patients in whom the iodide was used in cases of superficial ulceration of the fauces and mouth, 24 were cured, 8 benefited, and in 15 no good effect followed, and the author believes that mercury is of far greater value in these cases. In 27 cases of pustules of the skin, the cure was complete in 19, incomplete in 4, and in 4 the remedy was taken without effect,—these being fair grounds for inferring that it is a valuable medicine in cases of syphilitic ecthyma. In 21 cases of tubercle of the skin, 15 were cured, in 3 the cure was incomplete, in 3 the treatment was unsuccessful. Relapse was rare, and the author believes that the iodide is equally efficacious in tubercles as in pustules of the skin. Of 53 cases of rupia, 43 were completely cured, 7 incompletely, and in 3 only the remedy was useless. Thus the remedy is especially applicable in cases of rupia, and relapses are rare. In deep ulcers of the fauces the iodide was employed in 49 patients: 42 were cured, 3 benefited, 4 were not benefited, but were ultimately cured by the bichloride of mercury. It would appear that in these cases the iodide is equally beneficial whether mercury has or has not been previously employed. Relapses are rare, and when they occur, are in the first year. The iodide was employed in 3 cases of subcutaneous tubercle, in 2 successfully. Of 51 cases of tumours of the bones and periosteum, 8 were in men, 43 in women. In 6 only complete cure followed the use of the iodide, that is complete disappearance of the tumour and pain, in 22 the tumours were diminished in various degrees, in 23 they remained unaffected. The author has not made up his mind as to the value of the iodide in this class of cases, but believes that it is an efficacious remedy, *when compared with* others.

In pains of the bones (Dolores osteocopi,) in 73 patients, 58 again being females, 15 males, the pains altogether disappeared in 65 cases, in 3 diminished, and in 5 the iodide was given without effect. It would appear that the iodide is somewhat less efficacious in these cases when mercury has been previously taken, the recovery being less rapid. Relapses are rare, and generally occur within the first year, when they are observed. In no other symptom of syphilis, is the iodide so efficacious, and its effects so certain, as in these cases of pains in the osseous system, whether they occur by night or day, or have troubled the patient for years, or only for a few days. In 17 cases of caries and necrosis, 4 in men, 13 in women, the cure was complete in 6, incomplete in 4, and in 7 the medicine was useless; of these 7, 3 were afterwards cured by mercury. The author does not speak from personal experience of the efficacy of the iodide in cases of syphilitic cachexia—that state of emaciation and general disease of the whole organism we frequently observe without any prominent local symptoms, with languor, hectic, depression of spirits, and rapid decay of both mind and body, in which the iodide has been found most efficacious in this country.

We give some of the author's tables, as they are really of considerable value in the history of syphilis, and also may serve as a guide for the construction of somewhat similar accounts of various modes of treating other diseases.

The first table shows the comparative value of the iodide in a variety of cases.

| | No. of Cases. | Perfect cures. | Per centage of perfect cures. | Mean duration of disease. |
|---|---|---|---|---|
| Pains in the bones . . | 73 | 65 | 89·041 | 10·114 days. |
| Deep ulcers of the fauces . | 49 | 42 | 85·714 | 27·333 „ |
| Rupia . . . | 53 | 43 | 81·132 | 39·23 „ |
| Tubercle of the skin . . | 21 | 15 | 71·429 | 44·6 „ |
| Ecthymatous pustules of the skin . | 27 | 19 | 70·37 | 50·0 „ |
| Maculæ, squamæ, and papulæ of the skin | 49 | 26 | 53·061 | 57·731 „ |
| Superficial affections of the fauces | 47 | 24 | 51·064 | 40·583 „ |
| Caries and necrosis . . | 17 | 6 | 35·294 | 44·5 „ |
| Tumours of bones and periosteum | 51 | 6 | 11·765 | 36·167 „ |

The second table shows the comparative results of the treatment by the iodide when mercury had and had not been previously given.

| | No. of Cases. | Perfectly cured. | | Imperfectly cured. | | Treated without effect. | |
|---|---|---|---|---|---|---|---|
| | | With Mercury. | Without Mercury. | With Mercury. | Without Mercury. | With Mercury. | Without Mercury. |
| Maculæ, &c. | 22 | 11 | 2 | 1 | 2 | 3 | 3 |
| Pustules | 17 | 10 | 5 | 1 | 0 | 0 | 1 |
| Tubercles | 16 | 8 | 4 | 2 | 0 | 2 | 0 |
| Affections, superficial, of fauces, &c. | 34 | 13 | 5 | 2 | 3 | 4 | 7 |
| Rupia | 42 | 23 | 14 | 4 | 0 | 1 | 0 |
| Deep ulcer of fauces | 48 | 21 | 20 | 2 | 1 | 3 | 1 |
| Tumours of bone, &c. | 36 | 2 | 2 | 14 | 4 | 8 | 6 |
| Pains in bones | 68 | 35 | 17 | 2 | 0 | 3 | 1 |
| Caries and Necrosis | 14 | 2 | 1 | 4 | 0 | 5 | 2 |

The third table shows the results when the iodide was given alone, and when combined with the mercurial treatment.

| | No. of Cases. | Perfectly cured. | | Imperfectly cured. | | Treated without effect. | |
|---|---|---|---|---|---|---|---|
| | | With Mercury. | Without Mercury. | With Mercury. | Without Mercury. | With Mercury. | Without Mercury. |
| Maculæ, &c. | 44 | 7 | 17 | 3 | 6 | 7 | 4 |
| Pustules of skin | 27 | 4 | 15 | 0 | 4 | 2 | 2 |
| Tubercles of skin | 21 | 2 | 13 | I | 2 | 0 | 3 |
| Superficial affections of fauces | 46 | 3 | 21 | 2 | 6 | 4 | 10 |
| Rupia | 51 | 6 | 35 | 2 | 5 | 1 | 2 |

Another table follows on the number of relapses in each class of cases, but these we have already given in general terms, and we believe have presented our readers with all the important matter contained in this unpretending little essay. The author is evidently a careful inquirer and observer; he has recorded the result of his observations with much modesty and judgment, and thus written a work which must take a certain rank in the history of syphilis, and of the numerical system of medicine.

The work of Dr. Gauthier is a far less valuable and philosophical one, although it contains a great number of very interesting cases illustrating the efficacy of the treatment. He gives somewhat smaller doses of the medicine than Dr. Hassing, and always begins by a small dose and gradually increases it. The only general observation, however, which he has made which may be considered at all novel is that he has employed the iodide advantageously *sometimes*, he does not say how often, in the treatment of iritis; and he says that it has appeared to him to be particularly advantageous when the iritis has supervened, as is not unfrequently the case, during a course of mercurial treatment. He speaks of two cases in which, combined with bleeding and purgatives, good effects followed its administration, and refers to three similar ones, but allows that further observations are necessary before its value can be determined. As far as our experience goes, we may state that of all forms of secondary or tertiary syphilis, syphilitic iritis is the only one which has not been benefited by the iodide, and as calomel has such a marked effect over this form of iritis, especially when combined with albuminous effusion, we confess we should scarcely like to experiment upon the sight of a patient by a method of treatment which has the sole support of Dr. Gauthier's cases.

We have for upwards of four years been in the habit of prescribing the iodide very extensively in cases of secondary and tertiary forms of syphilis, and may give the result of our experience in a very few words. The best mode of administering it, we have found to be that recommended by Dr. Williams, eight grains three times a-day in camphor mixture. In some affections of the skin accompanied by much itching, also in certain cases of painful periosteal tumours, it has become more efficacious when given in decoction of sarsaparilla. The healing of the ulcers of rupia, and of the deep ulceration of the fauces are much accelerated by the local use of the ointment of the red oxide of mercury; in the latter cases being applied by a camel-hair brush. With these exceptions the medicine always acts much better if given alone: combination with mercury or any other drug, not only vitiates the experiment as to the value of the remedy, but also, as far as we have observed, retards the cure. We are convinced, from repeated trials, that there is something peculiar in the action of this particular dose, eight grains three times a-day; we have given it to young and old, rich and poor, feeble and weak, with invariable good effect. A smaller dose is seldom very efficacious, and a larger one is apt to produce coryza and headache, and lead to the necessity of discontinuing the medicine.

The fear of producing absorption of the testis or mammæ is perfectly groundless. Neither of the writers before us have ever seen it, nor have Ricord or Kluge who have given it very largely in the hospitals of Paris and Vienna. Dr. Gauthier says he has occasionally observed an eruption resembling acne or eczema depending upon the medicine; this we have never met with, nor with any other ill effect in the dose in which we now universally employ it. The coryza produced by a larger dose disappears spontaneously after the discontinuance of the medicine. Even in cases where local symptoms have not been strikingly benefited, the most marked improvement in the general health has resulted—the patients rapidly gaining flesh and strength, and losing the weary haggard look so remarkable in old syphilitic patients. We conclude by recommending our readers to follow our own practice, and give the iodide to every patient they meet with affected with secondary syphilis; in the great majority of cases it alone will effect the cure, and in the remainder will so improve the general health, as to render the patient fit to undergo any other course which the circumstance of his case may require.

It is curious to remark the very large proportion of females affected with affections of the osseous system, as compared with males, in the reports of Dr. Hassing. It would be well to inquire if the same fact has been observed in this country, and if so to what cause it could be attributed.

